# Chromosome Level Assembly of Homozygous Inbred Line ‘Wongyo 3115’ Facilitates the Construction of a High-Density Linkage Map and Identification of QTLs Associated With Fruit Firmness in Octoploid Strawberry (*Fragaria* × *ananassa*)

**DOI:** 10.3389/fpls.2021.696229

**Published:** 2021-07-14

**Authors:** Hye-Eun Lee, Abinaya Manivannan, Sun Yi Lee, Koeun Han, Jun-Geol Yeum, Jinkwan Jo, Jinhee Kim, Il Rae Rho, Ye-Rin Lee, Eun Su Lee, Byoung-Cheorl Kang, Do-Sun Kim

**Affiliations:** ^1^Vegetable Research Division, National Institute of Horticultural and Herbal Science, Rural Development Administration, Jeonju, South Korea; ^2^Department of Agriculture, Forestry and Bioresources, Plant Genomics and Breeding Institute, College of Agriculture and Life Sciences, Seoul National University, Seoul, South Korea; ^3^Department of Agronomy, Institute of Agriculture and Life Sciences, Gyeongsang National University, Jinju, South Korea

**Keywords:** *de novo* assembly, firmness, high density genetic map, homozygous inbred line, QTL analysis

## Abstract

Strawberry is an allo-octoploid crop with high genome heterozygosity and complexity, which hinders the sequencing and the assembly of the genome. However, in the present study, we have generated a chromosome level assembly of octoploid strawberry sourced from a highly homozygous inbred line ‘Wongyo 3115’, using long- and short-read sequencing technologies. The assembly of ‘Wongyo 3115’ produced 805.6 Mb of the genome with 323 contigs scaffolded into 208 scaffolds with an N50 of 27.3 Mb after further gap filling. The whole genome annotation resulted in 151,892 genes with a gene density of 188.52 (genes/Mb) and validation of a genome, using BUSCO analysis resulted in 94.10% complete BUSCOs. Firmness is one of the vital traits in strawberry, which facilitate the postharvest shelf-life qualities. The molecular and genetic mechanisms that contribute the firmness in strawberry remain unclear. We have constructed a high-density genetic map based on the ‘Wongyo 3115’ reference genome to identify loci associated with firmness in the present study. For the quantitative trait locus (QTL) identification, the ‘BS F_2_’ populations developed from two inbred lines were genotyped, using an Axiom 35K strawberry chip, and marker positions were analyzed based on the ‘Wongyo 3115’ genome. Genetic maps were constructed with 1,049 bin markers, spanning the 3,861 cM. Using firmness data of ‘BS F_2_’ obtained from 2 consecutive years, five QTLs were identified on chromosomes 3-3, 5-1, 6-1, and 6-4. Furthermore, we predicted the candidate genes associated with firmness in strawberries by utilizing transcriptome data and QTL information. Overall, we present the chromosome-level assembly and annotation of a homozygous octoploid strawberry inbred line and a linkage map constructed to identify QTLs associated with fruit firmness.

## Introduction

The octoploid strawberry (*Fragaria* × *ananassa*) is one of the most important horticultural crops worldwide. The genus *Fragaria* consists of 22 wild species with different ploidy levels, ranging from diploid (2*n* = 2*x* = 14) to decaploid (2*n* = 10*x* = 70). According to previous reports, the chromosomes of cultivated strawberry (2*n* = 8*x* = 56) evolved through a combination of polyploidy and repeated homoploid hybridization (Darrow, 1966; Whitaker et al., 2020). Owing to its pleasant aroma, flavor, antioxidant properties, and other vital health benefits, the consumption and the economic value of strawberry have been increasing (http://faostat.fao.org/site/567/). However, the complexity of the strawberry genome due to its high heterozygosity and polyploidy makes it difficult to implement molecular breeding approaches. In strawberry, before the availability of genomic resources, diagnostic molecular markers were developed and validated for the purpose of marker-assisted breeding or DNA-informed breeding (Iezzoni et al., 2020). Moreover, DNA tests aided the strawberry breeding programs by predicting desirable traits in parents and progenies (Oh et al., 2019). One of the major breeding initiatives “RosBREED” for Rosaceae crops like strawberry enabled the breeding of strawberry cultivars by bridging the available genetic and genomic resources for strawberry breeders (Iezzoni et al., 2010). The advent of diploid ancestor the *Fragaria vesca* reference genome (Shulaev et al., 2011) facilitated the strawberry breeding, particularly the construction of genetic linkage maps and quantitative trait locus (QTL) analysis in strawberry, which has been further enhanced by the recent availability of chromosome level assembly of the heterozygous octoploid strawberry ‘Camarosa’ (Edger et al., 2019). However, the availability of an additional reference genome sourced from highly homozygous cultivated strawberries can substantially benefit the identification of novel genes and genomic variations associated with vital traits and also can facilitate the genomics-based evolutionary studies in cultivated strawberries. Moreover, genetic variations associated with a trait of interest can be tailored to potential molecular markers that will aid in the marker-assisted selection in strawberries. According to previous reports, the availability of several high-quality genomes can be utilized for the discovery of a wide range of functional genomic variations by direct comparative analysis of the genomes (Chakraborty et al., 2018; Zhang et al., 2019). The presence of a single reference genome is insufficient for the investigation of copy number variants (CNVs) and presence/absence variants (PAVs) in plants (Golicz et al., 2016). Moreover, in crops like strawberry, the presence of an additional high-quality reference genome can enhance comparative genomics and pan-genome analysis.

The cultivated strawberry is highly heterozygous and polyploid, which poses numerous hindrances for genome assembly investigation. The first reference genome of *Fragaria vesca* ‘Hawaii-4’ was assembled, using combinations of short-read sequencing approaches, which resulted in an incomplete genome with 6.99% gaps (Shulaev et al., 2011). However, the improvement of *F. vesca* assembly, using a homozygous inbred line derived from S_7_ recombinant inbred line (RIL) and S_4_ derived RIL of ‘Hawaii-4’ by utilizing long-read PacBio single-molecule real-time sequencing and the BioNano optical map increased the contiguity of the assembly by 300 folds in comparison with the first version (Edger et al., 2018). Recently, Linsmith et al. (2019) have improved the assembly of *Pyrus communis* ‘Barrlet’ by producing a homozygous double-haploid line and utilization of long-read sequencing approaches. Similarly, the *de novo* assembly of *Malus domestica* ‘Golden Delicious’ double-haploid line was sequenced, using hybrid sequencing technologies, which incorporates both short- and long-read sequencing approaches to produce a high-quality reference genome arranged in 17 chromosomes (Daccord et al., 2017).

Hence, in the present study, we have utilized the highly homozygous strawberry inbred line ‘Wongyo 3115’ (S_9_ generation) developed from the ‘Benihoppe’ cultivar for the genome assembly. ‘Wongyo 3115’ produces pink-colored fruits with high firmness and a high sugar/acid ratio in comparison with other inbred lines. The homozygosity of ‘Wongyo 3115’ was investigated, using the genome-wide high-resolution melting-based SNP markers, and the results suggested that 96.6% of the genome in ‘Wongyo 3115’ is homozygous (Lee et al., 2020). The homozygous cultivar ‘Wongyo 3115’ can reduce complexity of the genome assembly process, and its ancestor ‘Benihoppe’ is a major cultivar of Japanese origin with various desirable characters (Mochizuki et al., 2013). Therefore, sequencing and assembly of highly homozygous octoploid strawberry inbred line ‘Wongyo 3115’, using PacBio single molecule real-time sequencing (SMRT), and short-read Illumina sequencing is reported.

Previously, linkage maps of cultivated octoploid strawberry (*F*. × *ananassa*) were constructed, using PCR-based markers like amplified fragment length polymorphism (AFLP) and simple sequence repeats (SSR) (Lerceteau-Köhler et al., 2003; Rousseau-Gueutin et al., 2008). In addition, linkage maps constructed, using PCR markers, displayed collinearity and synteny with the diploid strawberry (*F. vesca*) genome or wild strawberry-derived linkage maps (Sargent et al., 2009, 2012; Zorrilla-Fontanesi et al., 2011; Isobe et al., 2013; van Dijk et al., 2014). These linkage maps and markers were further utilized for QTL analysis of agronomic traits and metabolite contents (Zorrilla-Fontanesi et al., 2011; Lerceteau-Köhler et al., 2012; Labadie et al., 2020). However, due to the low-density of available maps and lack of genome and gene information in octoploid strawberry, there were limitations to propose sub-genomic location and candidate genes of identified QTLs. Therefore, the improvement of sequencing technology aided in the development of arrays based on the genomic information of diploid and octoploid strawberry sequencing data. Sequences of diploid and octoploid accessions were used for the development of Affymetrix IStraw90, IStraw35, 850K, and 50K Axiom arrays (Bassil et al., 2015; Verma et al., 2017a; Hardigan et al., 2020). Reduced genome sequencing methods, including GBS, ddRAD, and target-captured sequencing, identified a high number of SNPs in segregating F_1_ populations (Tennessen et al., 2014; Davik et al., 2015; Sánchez-Sevilla et al., 2015; Vining et al., 2017; Hossain et al., 2019). Using array- and sequencing-based approaches, QTLs controlling flowering behavior (Verma et al., 2017b), fruit quality (Alarfaj et al., 2021), and runner production (Hossain et al., 2019) were identified. High-density genetic maps successfully mapped QTLs within 2 Mbp, which shows the possibility to detect candidate genes. Hence, the high-density genetic map constructed in the present study will be beneficial for strawberry breeding.

Fruit firmness is considered an important polygenic trait among the strawberry breeders, which helps in lengthening the postharvest storage of strawberries. Firm fruits are considered for more extended storage and are less susceptible to pathogens than less firm strawberries (Dotto et al., 2006). Several factors such as cell wall organization, cuticle properties, ripening period, hormones, and environmental cues influence the firmness in strawberries (Chaïb et al., 2007; Saladié et al., 2007). Although the fruit firmness is attributed by several factors and enzyme activities, especially during the fruit ripening process, previous reports suggested the potential involvement of the expansin genes in the disassembly of the cell wall in strawberries (Dotto et al., 2006). In the present endeavor, we have constructed a genetic map and identified QTLs associated with firmness by utilizing the new assembly and annotation information of the homozygous octoploid strawberry ‘Wongyo 3115’. Furthermore, we have predicted potential candidate genes, including expansins associated with firmness, using the QTL information on octoploid strawberry.

## Results

### Genome Sequencing and Assembly of Homozygous Octoploid Strawberry ‘Wongyo 3115’

**Figure 1:**
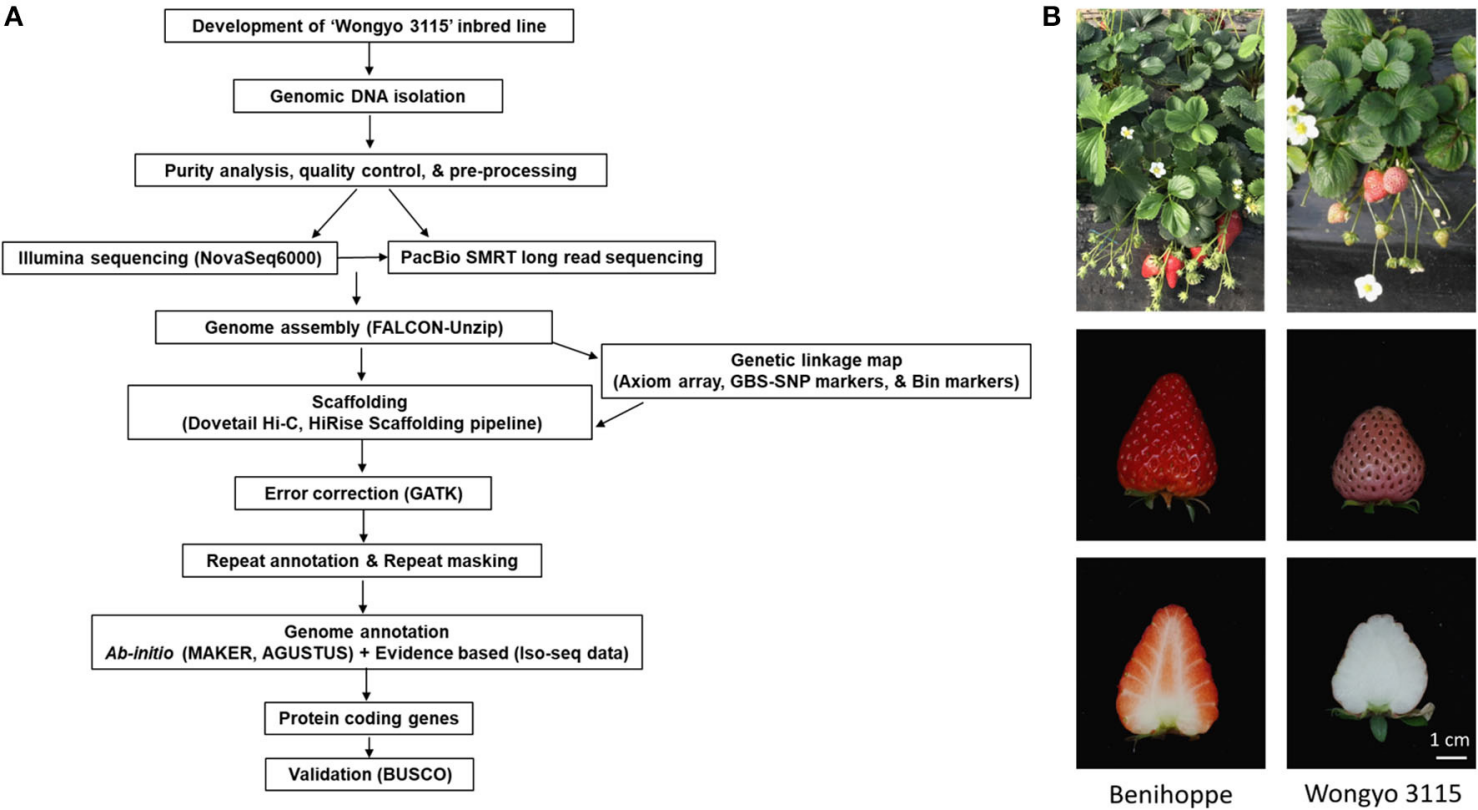
**(A)** An overview of the workflow pipeline employed in this study. **(B)** The phenotype of the ‘Wongyo 3115’ homozygous inbred line and its parent ‘Benihoppe’.

To perform a *de novo* genome assembly of ‘Wongyo 3115’ genome, we integrated two sequencing technologies, long-read PacBio and short-read Illumina (Figure 1A). The Illumina paired-end read data (17.1 Gb; Supplementary Table 1) were utilized for the estimation of genome size, correction, and evaluation of genome assembly. Based on the k-mer results, the ‘Wongyo 3115’ genome size was estimated, using the Jellyfish tool, which resulted in 788–804 Mb, which is consistent with the reported genome size of octoploid strawberry ‘Camarosa’ (Edger et al., 2019) (Supplementary Table 2; Supplementary Figure 1A). The k-mer results were utilized for the prediction of heterozygosity, using the GenomeScope tool (Supplementary Figure 1B) The present sequence data comprised of 22 × Illumina paired-end reads, 75.8 × PacBio Sequel long reads, and 95 × Hi-C reads. The PacBio SMRT sequencing resulted in a total of 4,132,073 PacBio subreads (Supplementary Table 3). The subread filtering generated 61.0 Gb of single-molecule sequencing data with mean read length of 14.7 kb and a maximum read length of 99.9 kb. The final reads were assembled into 323 primary contigs with an N50 value of 9.84 Mb and total length of 805.7 Mb. The assembly produced 844 haplotigs with the total length of 59.8 Mb and N50 of 75.5 kb (Supplementary Table 4). For the subsequent scaffolding process, the 323 primary contigs were employed. The hybrid scaffolding was performed, using HiRise software with Hi-C data to obtain assembly results at the scaffold level (Supplementary Figures 2, 3). The Dovetail Hi-C data (~76.5 Gb) improved the scaffolding of initial input assembly, consisting of 323 scaffolds to 208 final scaffolds with N50 of 27.3 and 805.6 Mb of genome length with 135 gaps (Table 1). The Hi-Rise resulted in 13 breaks in the input assembly with 133 numbers of joins (Supplementary Table 5). After hybrid scaffolding, PBjelly was employed for further gap filling.

**Table 1:** Assembly and annotation statistics of ‘Wongyo 3115’.

**Genomic features**	**‘Wongyo 3115’**
**Contig**
Number of contigs	323
Total size of contigs (bp)	805,664,111
N50 contig length (bp)	9,849,455
L50 contig count	29
GC contents (%)	39.35
**Scaffold**
Number of scaffolds	208
Total size of scaffolds (bp)	805,679,411
N50 scaffold length (bp)	27,373,183
L50 scaffold count	13
GC Contents (%)	39.35
**Genes**	151,892

Subsequently, a genetic linkage map was constructed for scaffold anchoring using, ‘BS F_2_ (I)’ population. The linkage map was generated, using the SNP markers obtained from genotyping-by-sequencing (GBS) and Axiom arrays. For the GBS analysis, a total of 140 lines of ‘BS F_2_ (I)’ population was used. A total of 5,791 markers, which are the sum of 4,936 Axiom markers and 855 GBS markers, were utilized for the linkage analysis and map construction. A total of 33 linkage groups were constructed with 1,245 bin markers (Supplementary Dataset 1). By integrating genetic map information, the ‘Wongyo 3115’ assembly was anchored and mapped to 28 pseudo-chromosomes (Supplementary Figure 4), consisting of 135 gaps with an estimated median gap length of 15.7 kb. The quality of genome was evaluated, using the consistency of physical and genetic maps that were constructed with representative SNP loci. Furthermore, the chromosomes were named in a similar manner to the recently published ‘Camarosa’ reference genome (Edger et al., 2019). In addition, the homozygosity of ‘Wongyo 3115’ and the recently sequenced ‘Camaroasa’ genome were compared by calling heterozygous SNPs obtained from Illumina reads of corresponding individuals (Supplementary Figure 5). Homozygous ‘Wongyo 3115’ showed fewer heterozygous SNPs than heterozygous ‘Camarosa’.

Gene prediction was performed, using *ab initio* approaches and evidence data as transcript and protein sequences. Gene annotations were made, using all protein sequences of the *Fragaria* genus. The gene models were further improved by providing MAKER with the IsoSeq data generated from the callus samples (Supplementary Table 6). Post annotation was performed to add putative gene functions and protein domains, using BLAST and InteProScan. Based on the mapping of 151,934 transcript sequences with a mean length of 218,274 bp onto the ‘Wongyo 3115’ genome assembly, we predicted a total of 151,892 genes with the gene density of 188.52 (gene/Mb) (Table 1). The gene ontology (GO) terms were assigned for the predicted transcript sequences and analyzed, using the BLAST2GO v.2.4 pipeline. Among the annotated genes, 40.6% were assigned with GO terms, and 56.1% were consigned with InterPro hits. Furthermore, Repeat modeler and Repeat Masker programs were employed to investigate the repeats in the ‘Wongyo 3115’ genome with the database search from DFAM and RepBase (Supplementary Table 7). Class I retrotransposons represented the largest transposable elements with 312.2 Mb, covering 37.25% of the sequenced genome, of which long terminal repeat (LTR) retrotransposons occupied 19.26%. In addition, the long interspersed nuclear elements (LINEs) and short interspersed nuclear elements (SINEs) represented 1.63% of the genome, and class II elements (DNA transposons) were 10.92%, and the unclassified repeats were identified as 5.44%. The final results displayed 6,985 tRNAs and 658 rRNAs, which include both 5S and 45S rRNAs. The location details of tRNAs and rRNAs present in the ‘Wongyo 3115’ assembly are provided in Supplementary Dataset 2.

### Validation of ‘Wongyo 3115’ Genome Assembly

For validation, we employed 1,440 gene sets of orthologs conserved in embryophyta (Supplementary Table 8). The results revealed that complete BUSCOs of 94.10% core genes/orthologs, complete and single copy BUSCOs (7.7%), complete and duplicated BUSCOs (86.4%), and fragmented BUSCOs (0.8) of embryophyta genes were present in the ‘Wongyo 3115’ genome.

To assess quality of our new reference genome, re-sequencing and mapping 10 strawberry cultivars widely cultivated in Asia and USA were performed. The re-sequencing of the strawberry cultivars, using the Illumina platform, resulted in an average of 47,856,088 bp reads with a length of 5,276,122,404 bp. After trimming, the high-quality reads were aligned to the ‘Wongyo 3115’ and ‘Camarosa’ reference genomes for the comparison of mapping alignment. The average mapping percentage of reads to the reference genomes produced similar mapping alignments with the ‘Wongyo 3115’ genome (89.85%) and the ‘Camarosa’ genome (87.49%) (Supplementary Table 9). Similarly, the average genome coverage of 91.61 and 91.01% was achieved for the ‘Wongyo 3115’ and ‘Camarosa’ genomes, respectively. The results suggest that the mapping efficiency of the reads to the ‘Wongyo 3115’ genome is in accordance with the recently published ‘Camarosa’ genome.

### Construction of a High-Density Genetic Linkage Map

Besides the 140 ‘BS F_2_ (I)’ lines used for genome assembly, additional 186 lines of ‘BS F_2_ (II)’ were genotyped, using the Axiom 35K strawberry chip, and used for QTL analysis of fruit firmness. A total of 6,494 markers displayed polymorphism between parental lines, and 5,527 markers were aligned to chromosomes and segregated in the F_2_ population (Supplementary Table 10). Segregation distortion was identified from some markers while most of the markers showed an expected segregation ratio (Supplementary Dataset 3). As the Axiom 35K strawberry chip was developed from the *F. vesca* genome, each marker aligned with more than one region by BLAST. Therefore, the bin map was constructed in two different methods to show chromosomal rearrangements of the reference genome and QTL analysis for fruit firmness.

**Figure 2:**
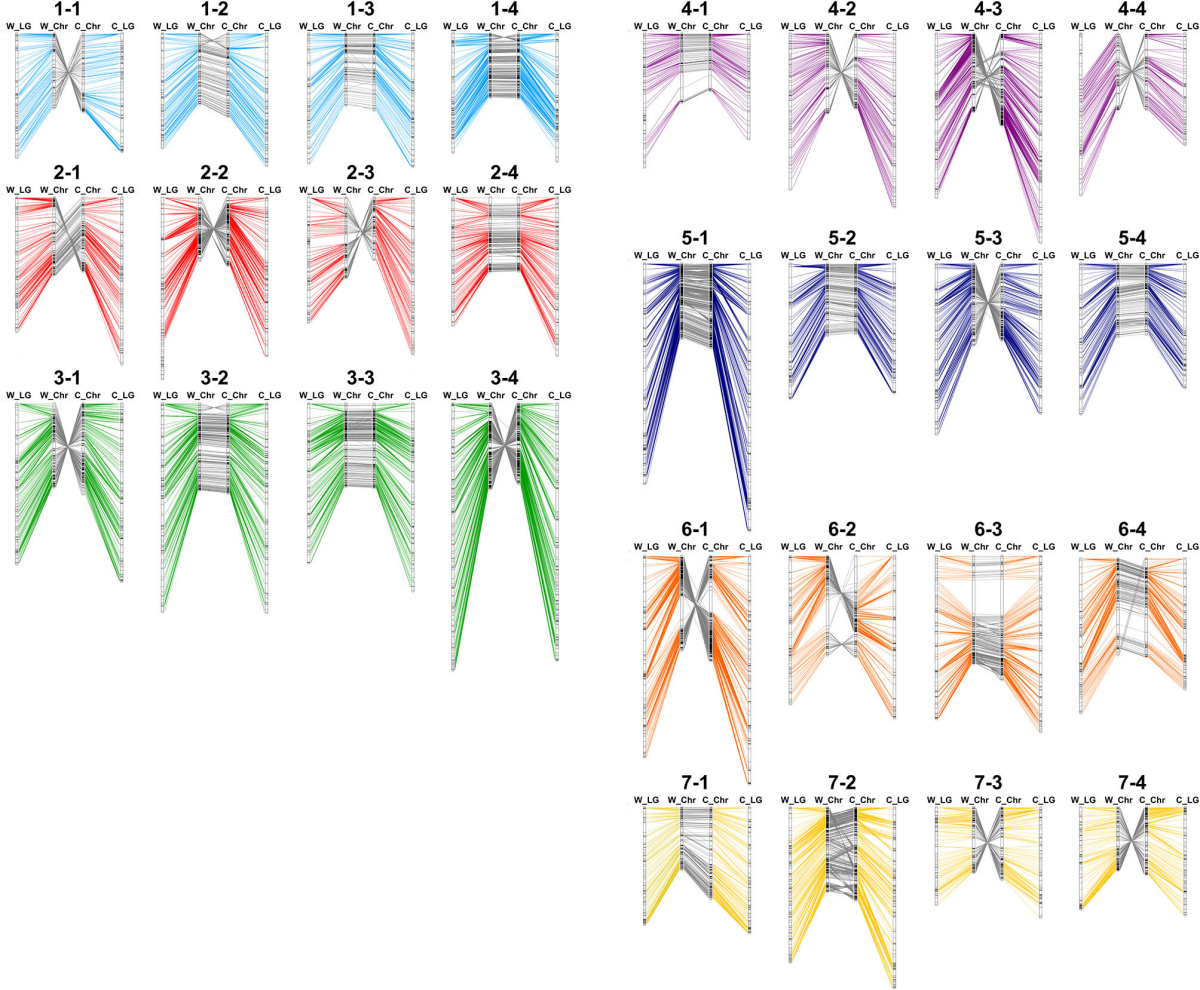
Comparison of physical and genetic position of markers in ‘Wongyo 3115’ and ‘Camarosa’ genomes. W_LG, the linkage group of ‘Wongyo 3115’; W_Chr, chromosome of ‘Wongyo 3115’; C_Chr, chromosome of ‘Camarosa’; C_LG; the linkage group of ‘Camarosa’.

First, to show the chromosomal rearrangements of the reference genome, genotypes of 2,586 markers were used repetitively two to six times for bin map construction in multiple sites. For example, flanking sequence of ‘AX-123356912’ was aligned with 14.27 Mbp of Chr1-2 and 15.21 Mbp of Chr1-3, and this marker was combined with two bin markers on Chr1-2 and Chr1-3. By the sliding window approach, a genetic map of 4,961 cM was constructed with 2,697 bin markers (Supplementary Dataset 3). Similarly, a 5,186-cM bin map was constructed based on the ‘Camarosa’ genome with 2,768 bin markers. Genetic and physical positions of bin markers were colinear in both the ‘Wongyo 3115’ and ‘Camarosa’ genomes (Figure 2). Furthermore, the Axiom markers used for the construction of the bin map were employed to analyze synteny between ‘Wongyo 3115’ and ‘Camarosa’ genomes. The constructed map showed high synteny between two genomes. However, inversions were detected on chromosomes 1-2, 1-4, 3-2, and 6-2. Low collinearity between linkage groups and the ‘Camarosa’ genome on chromosomes 1-2 and 6-2 was also detected from 140 ‘BS F_2_ (II)’ bin maps. In addition, chromosomes, such as 1-1, 2-2, 2-3, 3-1, 3-4, 4-2, 4-3, 4-4, 5-3, 6-1, 7-3, and 7-4 displayed reverse orientation.

Furthermore, we compared the ‘Wongyo 3115’ physical map with previously reported octoploid strawberry genetic maps (Supplementary Dataset 4). Linkage groups of ‘Redgauntlet’ × ‘Hapil’ F_1_ (Alarfaj et al., 2021) matched with individual chromosomes, while some of the linkage groups displayed breakage on ‘Sulhyang’ × ‘Senga-sengana’ F_1_ (Lee et al., 2020) and ‘232’ × ‘1,392’ F_1_ (Sánchez-Sevilla et al., 2015). All the linkage groups matched with chromosomes 1-2, 1-4, 3-2, and 6-2 showed collinearity between genetic position and physical position in ‘Wongyo 3115’, except the linkage group LG6.4 of ‘Redgauntlet’ × ‘Hapil’ F_1_ and LG1-1 of ‘Sulhyang’ × ‘Senga-sengana’ F_1_ aligned with Chr6-2 and 1-4, respectively (Supplementary Figure 6).

For QTL analysis, each axiom marker were used only once to construct a bin map to reduce mapping errors. The bin map was constructed after chromosomal locations of axiom markers were determined by linkage mapping. Due to the low density of markers in multiple chromosomal regions, 28 pseudomolecules were divided into 44 linkage groups with the total genetic map size of 3,861 cM (Supplementary Dataset 3; Supplementary Figure 7). In addition, to validate the genetic map, QTL mapping for fruit core color was performed, and the *MYB10* gene controlling fruit and flesh color of strawberry reported by Castillejo et al. (2020) was mapped on the QTL region (Manivannan et al., unpublished).

### Identification of QTLs Associated With Firmness in the ‘BS F_2_ (II)’ Population

**Figure 3:**
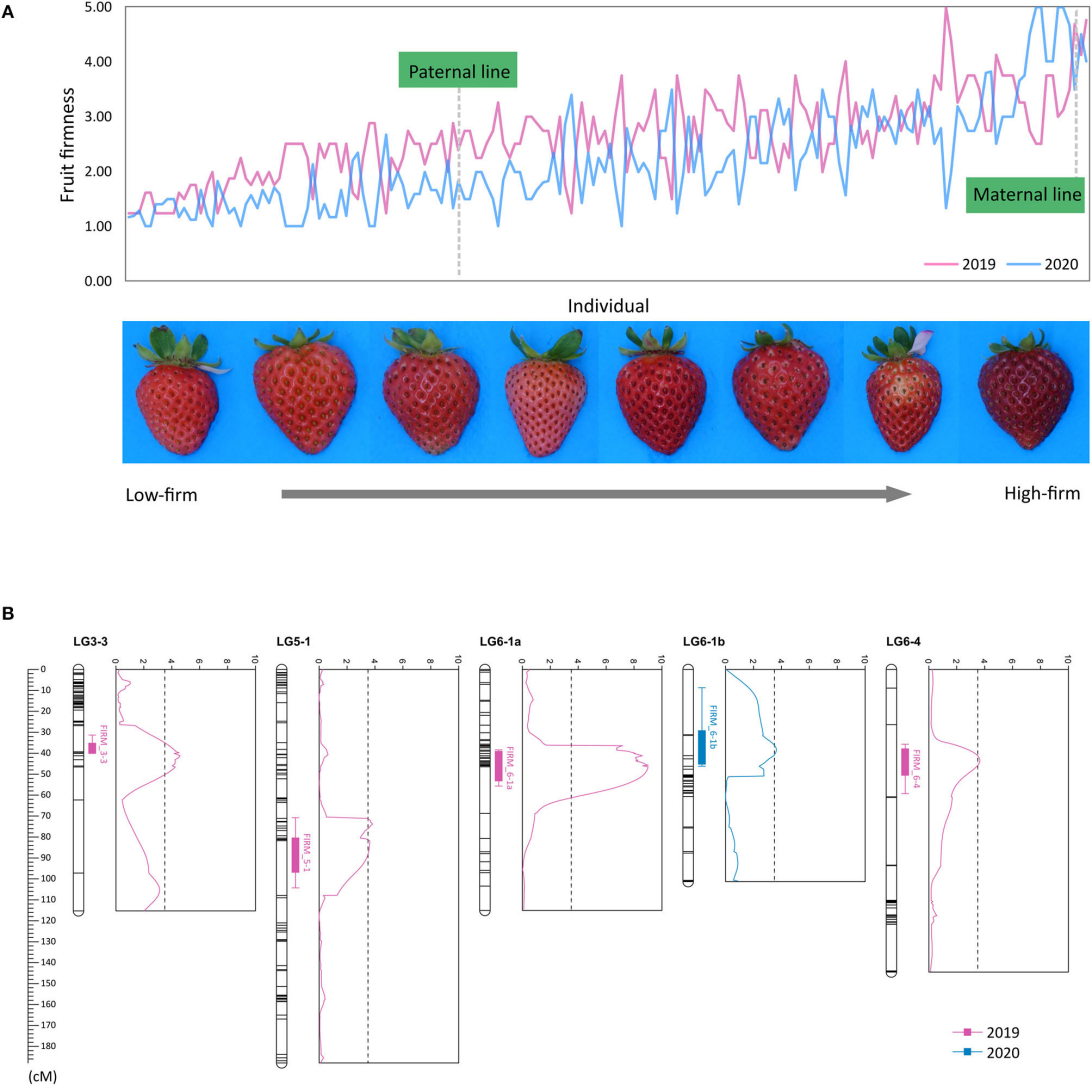
**(A)** Distribution of fruit firmness in ‘BS F_2_(II)’ evaluated in (a) 2019 and (b) 2020. **(B)** Positions of five quantitative trait locus (QTLs) associated with firmness marked in the genetic map, along with the logarithm of the odds (LOD) graph.

Fruit firmness of the ‘BS F_2_ (II)’ population was evaluated as five scales. The maternal line was firmer than the paternal line, and most of the F_2_ individuals were softer than the maternal line (Figure 3A). QTLs controlling fruit firmness were analyzed, using a bin map of ‘Wongyo 3115’ and phenotype data evaluated in 2 consecutive years (2019 and 2020) (Table 2). A total of five QTLs associated with firmness were detected on chromosomes 3-3, 5-1, 6-1, and 6-4 (Figure 3B). Among the QTLs, one QTL was detected on chromosome 6-1 based on the phenotype data obtained in the year 2019. The other four QTLs detected from 2020 phenotype data illustrated 49% of the total variation of the phenotype, while no QTL was detected commonly in 2 years. The QTL information and ‘Wongyo 3115’ annotation data were utilized to predict candidate genes associated with firmness. To investigate the candidate genes associated with fruit firmness, the genes that displayed differential expression between the high-firm inbred line ‘Wongyo 3115’ and the low-firm inbred line ‘P69’ based on the transcriptome analysis and synergistically located in QTL regions were selected. The QTL regions covered a total of 408 differentially expressed genes based on the physical position details retrieved from the ‘Wongyo 3115’ annotation data. Among the QTL regions, *FIRM_6-1a* consisted of a higher number of genes (115) followed by *FIRM_6-4* (91 genes). The QTLs, *FIRM_5-1, FIRM_6-1b*, and *FIRM_3-3* encompassed 90, 65, and 47 genes, respectively (Supplementary Dataset 5).

**Table 2:** QTLs for fruit firmness detected in 2 years.

**QTL**	**Chr**	**Position (cM)**	**Physical position (Mbp)**	**LOD**	**R^**2**^ (%)**	**Additive effect**	**Dominant effect**	**Year**
*FIRM_3-3*	3-3	31.3–40.1	22.0–24.3	4.53	8.81	−0.38	0.03	2020
*FIRM_5-1*	5-1	70.8–104.3	10.8–15.6	3.83	9.15	0.35	−0.13	2020
*FIRM_6-1a*	6-1	38.4–55.7	5.5–9.4	9.01	23.48	0.49	−0.31	2020
*FIRM_6-1b*	6-1	8.7–46.2	24.5–26.1	3.64	10.73	−0.32	0.10	2019
*FIRM_6-4*	6-4	35.7–59.3	4.6–7.4	3.66	0.00	0.69	−0.84	2020

**Figure 4:**
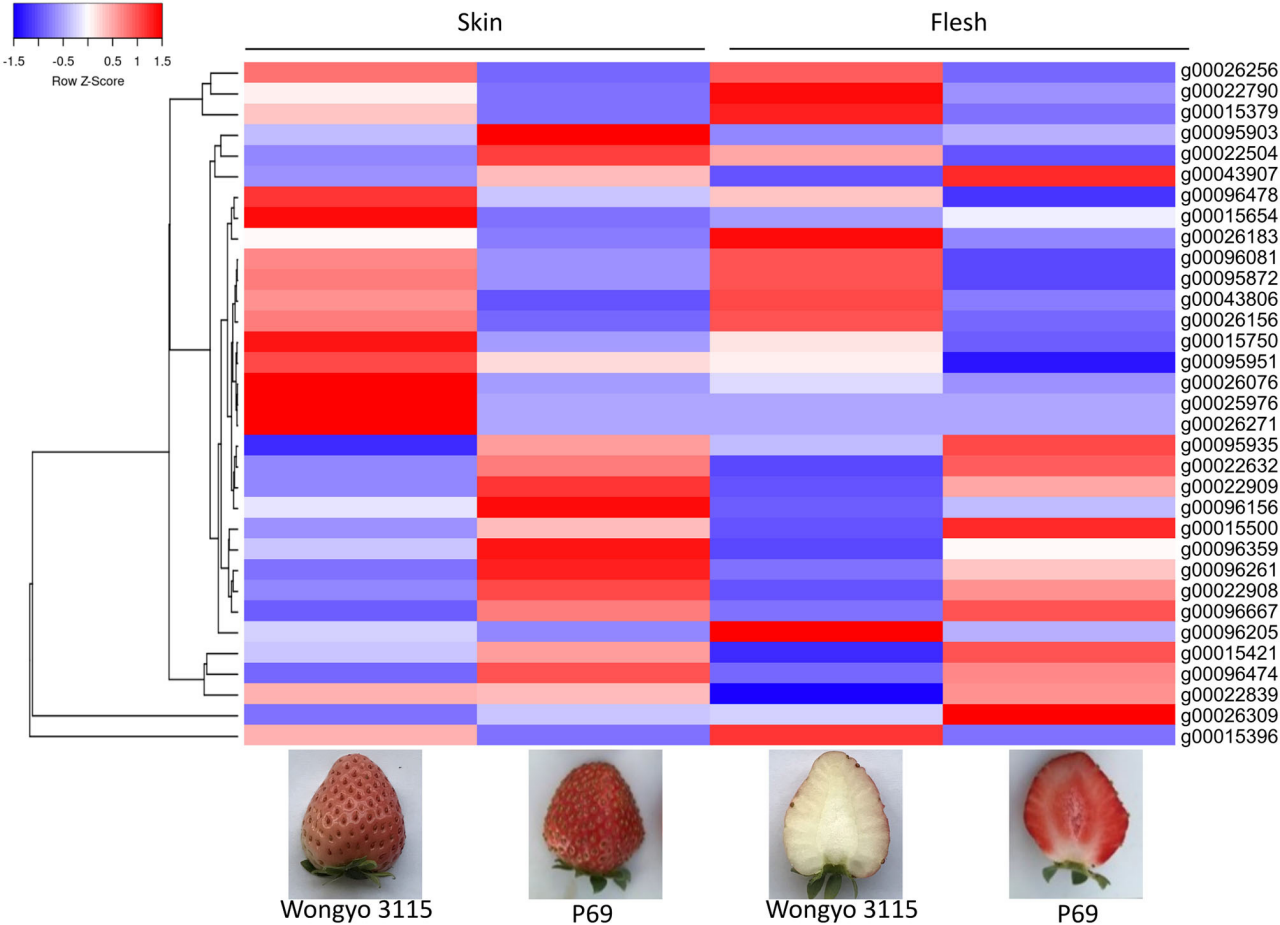
Heat map representation of 33 differentially expressed predicted candidate genes associated with fruit firmness.

The paired analysis between two inbred lines was performed with the filtering criteria of 2-fold-change difference. The number of differentially expressed genes (DEGs) only in skin and flesh were 70 and 83, respectively. In contrast, a total of 255 genes showed a difference in the expression in both tissues. Furthermore, the differentially expressed genes located in the QTL regions were extracted based on the physical position from the annotation data of ‘Wongyo 3115’ assembly. A total of 33 potential candidate genes, including vital transcription factors associated with firmness, have been identified by utilizing the transcriptome and QTL data (Tables 3, 4, Figure 4, Supplementary Dataset 5). Particularly, we identified the expansin gene (*EXPA3)*, pectin acetylesterase 8 (*PAE8*), pectate lyase4, ß-galactosidase 5 (*BGAL5*), and genes involved in auxin metabolism in the QTL regions, which play a vital role in the determination of firmness in strawberries. In addition, the transcriptome analysis indicated the upregulation of *EXPA3, PAE8*, and pectate lyase4 in the low firm ‘P69’ inbred line. However, the *BGAL5* displayed a high-abundance level in the high firm inbred line ‘Wongyo 3115’. Taken together, candidate genes predicted in the QTL regions could influence fruit firmness; however, the exact molecular rationale behind these genes in firmness has to be investigated in the future.

**Table 3:** Predicted candidate genes in QTLs associated with the firmness in the ‘BS F_2_ (II)’ population.

**QTL**	**Gene id**	**Chromosome**	**Description**	**Function**
*FIRM_6-1b*	g00026256	6-1	AGPS1: Glucose-1-phosphate adenylyltransferase	Starch metabolism
*FIRM_6-1a*	g00022909	6-1	pgi: Glucose-6-phosphate isomerase	
*FIRM_5-1*	g00095935	5-1	Fructose-1-6-bisphosphatase	
*FIRM_6-1b*	g00026183	6-1	PGK1: Phosphoglycerate kinase 1	
*FIRM_6-1a*	g00022504	6-1	EXPA3: Expansin-A3	Cell wall biogenesis
*FIRM_5-1*	g00096667	5-1	PAE8: Pectin acetylesterase 8	/organization
*FIRM_6-1a*	g00022632	6-1	At1g30350: Probable pectate lyase 4	
*FIRM_6-1b*	g00026309	6-1	FLA2: Fasciclin-like arabinogalactan protein	
*FIRM_3-3*	g00043907	3-3	ADF1: Actin-depolymerizing factor 1	
*FIRM_5-1*	g00096474	5-1	XTH30: Probable xyloglucan endotransglucosylase/hydrolase protein 30	
*FIRM_6-4*	g00015379	6-4	BGAL5: Beta-galactosidase 5	
*FIRM_6-1b*	g00025976	6-1	TSB2: Tryptophan synthase	Auxin metabolism
*FIRM_5-1*	g00096359	5-1	ARG7: Indole-3-acetic acid-induced protein ARG7	
*FIRM_6-4*	g00015654	6-4	LAX2: Auxin transporter-like protein 2	
*FIRM_5-1*	g00096205	5-1	NAC071: NAC domain-containing protein 71	
*FIRM_6-1a*	g00022839	6-1	PAL1: Phenylalanine ammonia-lyase 1	Secondary metabolism/
*FIRM_6-4*	g00015421	6-4	DFR: Bifunctional dihydroflavonol 4-reductase/flavanone 4-reductase	Lignin biosynthesis
*FIRM_6-1a*	g00022908	6-1	4CLL7: 4-coumarate–CoA ligase-like 7	
*FIRM_5-1*	g00096156	5-1	PER20: Peroxidase 20	
*FIRM_6-1b*	g00026271	6-1	CAD1: Probable cinnamyl alcohol dehydrogenase 1	
*FIRM_5-1*	g00095951	5-1	HST: Shikimate O-hydroxycinnamoyltransferase	
*FIRM_6-1a*	g00022790	6-1	PIP2-1: Aquaporin PIP2-1	Membrane transportation
*FIRM_6-1b*	g00026156	6-1	CYP97B2: Cytochrome P450 97B2	Lipid biosynthesis
*FIRM_6-4*	g00015750	6-4	UGT85A5: UDP-glycosyltransferase 85A5	Defense response/
*FIRM_3-3*	g00043806	3-3	Chitinase 2	glucosinolate biosynthesis

**Table 4:** Predicted candidate regulatory genes in QTLs associated with the firmness in the ‘BS F_2_ (II)’ population.

**QTL**	**Gene id**	**Chromosome**	**Description**	**Function**
*FIRM_5-1*	g00095872	5-1	HAT4: Homeobox-leucine zipper protein HAT4	DNA binding
*FIRM_5-1*	g00096478	5-1	BZIP16: bZIP transcription factor 16	
*FIRM_6-4*	g00015396	6-4	BZIP44: bZIP transcription factor 44	
*FIRM_6-4*	g00015500	6-4	WRKY33: Probable WRKY transcription factor 33	
*FIRM_5-1*	g00095903	5-1	ERF5: Ethylene-responsive transcription factor 5	
*FIRM_5-1*	g00096261	5-1	MYC4: Transcription factor MYC4	
*FIRM_5-1*	g00096081	5-1	FLZ2: FCS-Like Zinc finger 2	
*FIRM_6-1b*	g00026076	6-1	PIL13: Transcription factor PHYTOCHROME INTERACTING FACTOR-LIKE 13	

## Discussion

Homozygosity is vital for the assembly of the genome with accuracy, especially in polyploids like strawberry. The heterozygous regions in the polyploids tend to complicate assembling the genome and cause difficulties in haplotype phasing (Schatz et al., 2012; Nowak et al., 2015). Genome sequencing and chromosome level assembly of octoploid strawberry are laborious processes due to their high heterozygosity and ploidy levels. However, recently, Edger et al. (2019) have successfully generated the whole genome assembly of the heterozygous strawberry ‘Camarosa’ and determined the phylogenomic relationship among the progenitors of octoploid strawberry. To identify better candidate genes for molecular breeding and gain deeper insights into structural variations, the availability of multiple reference genomes is necessary, particularly in crops like strawberry. Therefore, in the present endeavor, we have assembled and annotated the chromosome level assembly of the highly homozygous strawberry inbred line ‘Wongyo 3115’.

The homozygosity of ‘Wongyo 3115’ has been validated, using the SNP markers in our previous report (Lee et al., 2020). Our assembly strategy incorporated the hybrid approaches with short- and long-read sequencing technologies to acquire the promising genome sequence assembly. Incorporating Illumina-based short-read sequences for the error correction on long-read PacBio SMRT-based sequencing can greatly improve the reference genome assembly quality (Zhang et al., 2018; Chen et al., 2019; Hu et al., 2019). Moreover, the high homozygosity of the inbred line facilitated the sequencing of the complex cultivated strawberry genome. This is the first report of whole-genome sequencing of the octoploid strawberry inbred line with high homozygosity. The estimated genome size of ‘Wongyo 3115’ is 804 Mb, which is consistent with the estimated genome size of the recently sequenced ‘Camarosa’ genome. Similarly, the final assembly of ‘Wongyo 3115’ consisted of 805.6 Mb, which is in accordance with the assembly length of ‘Camarosa’ (805.4 Mb) (Edger et al., 2019). The homozygous strawberry reference genome with only 135 gaps (N50: 26,750 bp) can provide an excellent resource for the genome-wide study of various strawberry cultivars.

The ‘Wongyo 3115’ genome consisted of 151,892 genes, 151,934 transcripts, and 598,688 CDSs, whereas 108,087 protein-coding genes were annotated in ‘Camarosa’ (Edger et al., 2019). The increased number of genes in the ‘Wongyo 3115’ genome could have resulted from the difference in the tissue employed for the transcriptome data generation and implementation of default parameters for the gene prediction. In addition, a difference in the gene numbers could have also been resulted due to the difference in gene content of the accessory genome. For instance, the gain or loss of genes located in the tandem repeat regions of the genome during the unequal crossing-over could also lead to the difference in number of genes. The repetitive sequences majorly constitute the eukaryotic genome and encompass various vital functions that determine the chromosomal rearrangements, regulation of gene expression, and evolution of the genome. The present ‘Wongyo 3115’ genome consisted of 312 Mb of repetitive sequence, which accounted for 38.75% of the assembled genome. Similarly, the ‘Camarosa’ genome consisted of 36% repetitive sequence with the majority of the repeats contributed by LTR_RT transposons (Edger et al., 2019). The evaluation of the genome assembly, using BUSCO, illustrated the presence of 94.10% core genes/orthologs that determine the completeness of the genome. Furthermore, we performed re-sequencing of 10 widely cultivated strawberry cultivars to validate the continuity and completeness of ‘Wongyo 3115’ assembly, using the read alignment ratio. The results suggested that the highest average percentage of mapped reads (89.85%) was achieved with ‘Wongyo 3115’ assembly for the re-sequenced cultivars. The comparison of re-sequenced mapped reads percentage and genome coverage of ‘Wongyo 3115’ displayed high similarity with the recently published ‘Camarosa’ genome. This illustrated the completeness of ‘Wongyo 3115’ assembly. Furthermore, ‘Wongyo 3115’ assembly and annotation information were utilized to construct a high-density genetic linkage map and identify QTLs associated with firmness.

In this study, we employed multiple genotyping methods to construct a high-density genetic map of the F_2_ population. This is the first genetic map of the F_2_ population of cultivated octoploid strawberry (*F*. × *ananassa*) derived from inbred lines. Maternal and paternal parents of the F_2_ population are inbred lines from the cultivars ‘Benihoppe’ and ‘Sachinoka’, respectively (Lee and Lee, 2017). ‘BS F_2_ (I)’ was genotyped, using GBS and IStraw90 array. Missing data from GBS and array were imputed by the sliding window approach, and bin maps were constructed and used for chromosome assembly and QTL analysis. By comparative mapping of ‘Wongyo 3115’ and the ‘Camarosa’ genome, possible scaffolding errors were detected on chromosomes 1-2, 1-4, 3-2, and 6-2 (Figure 2). In previous research, scaffolding errors on chromosomes 1-2, 1-4, 2-1, 2-3, and 6-2 were also supported with the wild octoploid genetic maps (Hardigan et al., 2020). Large rearrangement patterns on chromosome 1-2, 1-4 and 6-2 are similar with ‘BS F_2_ (II)’, ‘PI552277’, ‘PI61243’, and ‘Del Norte’ genetic maps, which show that the ‘BS F_2_ (II)’ genetic map can be used for correction of scaffolding errors in the ‘Camarosa’ genome.

Furthermore, the bin map of ‘BS F_2_ (II)’ was employed to identify QTLs associated with fruit firmness. Multiple QTLs for fruit firmness evaluated by penetrometer were identified, using low-density linkage maps in strawberry (Zorrilla-Fontanesi et al., 2011; Lerceteau-Köhler et al., 2012). Similarly, Verma et al. (2017b) analyzed QTL for fruit quality-related traits, including fruit firmness with nondestructive methods, using array-based high-density linkage maps (Bassil et al., 2015). In addition, Antanaviciute et al. (2017) used a fruit firmness tester and mapped eight QTLs in a high-density bin map. By comparison with previously reported expansin genes, the *FaEXP2* gene was located in the QTL region on linkage group 2C (Antanaviciute, 2016). Expansins are considered as excellent target genes for the investigation of firmness in strawberries. In general, expansins break the hydrogen bonds between cellulose microfibrils and cell wall matrix polysaccharides, increasing the cell wall permeability to several hydrolases (McQueen-Mason and Cosgrove, 1995; Rose et al., 1997; Rose and Bennett, 1999).

In a similar manner, Molina-Hidalgo et al. (2013) employed a nondestructive firmness test and detected a putative rhamnogalacturonate lyase gene (*FaRGlyase1*) in a minor QTL [logarithm of the odds (LOD) < 2.0] region, which was proposed to involve in cell-wall degradation. Based on the physical position of QTL-linked markers in the ‘Wongyo 3115’ genome, we compared the previously identified QTLs with the QTLs detected in this study. However, no QTL or candidate gene was co-localized. In addition, the QTLs detected from this study were year dependent, which shows that the fruit firmness of strawberry is highly affected by environment and controlled by multiple genetic factors. Therefore, to verify the significance of detected QTLs and to understand the mechanism controlling strawberry fruit firmness, additional QTL analysis in multiple genetic populations or association study would be helpful.

The annotation data of ‘Wongyo 3115’ and QTL information were utilized to predict potential candidate genes that could influence the firmness in strawberries. These genes were involved in various essential processes, such as starch metabolism, cell wall organization, auxin metabolism, secondary metabolism, lignin biosynthesis, and transcription regulation. Potential genes, such as *EXPA3, PAE8*, pectate lyase4, *BGAL5, FLA2*: Fasciclin-like arabinogalactan protein, *ADF1*: Actin-depolymerizing factor 1, *XTH30*: Probable xyloglucan endotransglucosylase/hydrolase, with an active role in cell wall organization, have been identified as candidate genes in *FIRM_6-1, FIRM_6-4, FIRM_3-3, and FIRM_5-1* QTL regions. Previous reports evidenced the role of expansins in strawberry fruit ripening and softening of fruit texture (Dotto et al., 2006; Valenzuela-Riffo and Morales-Quintana, 2020). The expression of various expansins during the ripening process has been reported in several fruits such as strawberry (Civello et al., 1999), tomato (Rose and Bennett, 1999), pear (Hiwasa et al., 2003), banana (Asha et al., 2007), apple (Goulao et al., 2008), and grape (Dal Santo et al., 2013). In papaya, the expression of *EXPA* during ripening has been influenced by plant hormone-based regulation of α-expansins in softening of fruits (Gaete-Eastman et al., 2009). Similarly, Valenzuela-Riffo and Morales-Quintana (2020) illustrated the binding mechanism of *EXPA2* in strawberry cultivars during fruit softening. According to Wu et al. (2021), the pectin acetylesterase gene enhanced the fruit softening in apple; similarly, in our results, the low firm ‘P69’ consisted of a higher expression level of pectin acetylesterase. Moreover, in strawberry, the silencing of pectate lyase and ß-galactosidase enhanced fruit firmness by influencing pectin metabolism (Salentijn et al., 2003; Paniagua et al., 2016). The xyloglucan endotransglucosylase/hydrolase (XTH) is involved in the cell wall modification by catalyzing the endolytic disintegration of xyluglucan polymers and binding of newly formed xyloglucans. In strawberry, the higher expression of XTH results in fruit softening (Witasari et al., 2019); in our results, the low firm ‘P69’ consisted of higher levels of XTH in comparison with high firm ‘Wongyo 3115’.

Furthermore, the *FIRM_6-1a* QTL region encompassed the PIP2-1 aquaporin gene; in strawberry, higher expression of fruit specific aquaporin in high-firm strawberry cultivar ‘Camarosa’ in comparison with the low-firm ‘Toyonoka’ has been reported (Alleva et al., 2010). According to Alleva et al. (2010), the fruit-specific PIP aquaporins play a vital role in the regulation of water transport based on their expression levels, and their participation in fruit ripening, coupled with softening in strawberries, has been investigated. However, the investigation of aquaporins in firmness-associated studies in strawberries will enhance the understanding of fruit development in strawberries. In the present study, QTL regions, such as *FIRM_5-1, FIRM_6-4*, and *FIRM_6-1b* consisted of predicted regulatory genes. Recently, the importance of regulatory genes in the development of strawberry fruits and the possible molecular mechanism behind the firmness has been investigated (Vallarino et al., 2020). Several studies insisted the potential role of hormones and transcription factors in the development of fruit (Aharoni and O'Connell, 2002; Pillet et al., 2015; Hartl et al., 2017; Sánchez-Sevilla et al., 2017). In the future, the potential role of TFs associated with the firmness trait can be achieved, and the present QTL data can aid in the process.

### Conclusion

In the present endeavor, we presented a chromosome-level assembly of the highly homozygous octoploid strawberry ‘Wongyo 3115’ genome, utilizing long- and short-read sequencing approaches. Furthermore, the ‘Wongyo 3115’ genome data were employed for the construction of the genetic linkage map to identify QTLs associated with strawberry fruit firmness. The QTL regions encompassed vital candidate genes that influence firmness in strawberry. Furthermore, we investigated the expressions of candidate genes in the transcriptome of skin and flesh tissues of high-firm and low-firm strawberry inbred lines. Overall, the highly homozygous ‘Wongyo 3115’ genome can accelerate the genomic and genetic research focused on SNP discovery, gene discovery, genetic mapping, and genome-wide association studies on octoploid strawberries.

## Materials and Methods

### Plant Materials

To overcome the complexity of the octoploid strawberry genome, we used the homozygous inbred line ‘Wongyo 3115’ (Figure 1B) (application number 2014-152, Korea Seed and Variety Service) for *de novo* genome assembly. The ‘Wongyo 3115’ has conical type fruit with strong firmness, pink skin, and white flesh. It was developed from the Japanese cultivar ‘Benihoppe’ by self-pollination for nine generations (S9) at the National Institute of Horticultural and Herbal Science, Jeonju, South Korea (Jeong et al., 2015) (Supplementary Figure 8). In order to construct the genetic linkage map, an inbred line ‘8-10’ derived from ‘Benihoppe’ and 105 (14-9) derived from ‘Sachinoka’ were crossed, and two F_2_ populations with different individuals were developed (Supplementary Figure 9). A total of 140 F_2_ [‘BS F_2_ (I)’] and 186 F_2_ [‘BS F_2_ (II)’] were employed for genome assembly and QTL analysis, respectively. Furthermore, to assess the quality of our new reference sequence, 10 strawberry cultivars widely cultivated in Asia and USA have been selected for re-sequencing (Supplementary Table 9). For the fruit firmness study, high-firm ‘Wongyo 3115’ and low-firm inbred line ‘P69’ derived from the ‘Benihoppe’ cultivar were employed for the transcriptome analysis. The firmness was estimated by a nondestructive method (Mathey et al., 2013; Verma et al., 2017b). Fruit firmness was scored by compressing 80–0% ripe fruit between a thumb and a forefinger and scored from mushy to hard (Scored 1 to 5, level 1: very soft; level 5: very firm; Supplementary Figure 8).

### Genome Sequencing and Assembly

#### Genome Size Estimation

To estimate the size of the genome, genomic DNA was isolated from the tender leaves of ‘Wongyo 3115’ by the CTAB method (Lee et al., 2020). An Illumina paired-end library of 350 bp was constructed according to the Illumina Truseq Nano DNA Library prep protocol and sequenced in Illumina NovaSeq 6000 system (Illumina, USA). Illumina read data were used for the estimation of genome size, correction and evaluation of the genome assembly. To estimate the genome size, we used the whole genome sequencing data, k-mer counting by Jellyfish version 2.1.3 with the k-mer size set to 17, 19, and 25. The genome size was estimated, using the following formula: genome size = total number of nucleotides/peak depth of k-mer frequency distribution (Marçais and Kingsford, 2011). In addition, the GenomeScope (http://qb.cshl.edu/genomescope/) was employed to obtain estimates for genome sizes, heterozygosity, and duplication levels.

#### PacBio SMRT Sequencing

Genomic DNA were extracted from tender leaves of ‘Wongyo 3115’, using the CTAB method (Lee et al., 2020) and fragmented into 20 kb, using a g-TUBE (Covaris, USA). Furthermore, the fragments were purified, using AMpureXP bead purification system to remove the small fragments. After purification, the SMRTbell library was constructed, using SMRTbell™ Template Prep Kit 1.0 (PN 100-259-100), and the BluePippin Size selection system was employed to remove the small fragments for a large-insert library. Using Sequel Binding Kit (2.0), sequencing primer and DNA polymerase were bound to the SMRTbell library, and the complex was purified with SMRTbell Clean-up columns (SMRTbell® Clean Up Columns v2 Kit-Mag: PN 01-303-600). The MagBead Kit (Pacific Biosciences) was used to bind the library complex with MagBeads before sequencing. The polymerase-SMRTbell-adaptor complex was then loaded into zero-mode waveguides (ZMWs). The SMRTbell library was sequenced, using 25 SMRT cells (Pacific Biosciences, Sequel™ SMRT® Cell 1M v2) with Sequel Sequencing Kit (2.1), and 1 × 600-min movies were captured for each SMRT cell, using the Sequel (Pacific Biosciences)-sequencing platform. Finally, the resulting Sequel raw bam files were converted into subreads in the FASTA format, using the standard PacBio SMRT Link v10.1 software package.

#### Dovetail Hi-C Library Preparation

The dovetail Hi-C library was prepared according to the instructions of the manufacturer (Dovetail Hi-C Library kit). Young leaves were homogenized, and.25 mg of plant tissue was cross-linked with PBS/formaldehyde and then chromatin prepared with SDS and a wash buffer. After normalizing the chromatin plant sample, 800 ng of chromatin was used for the library construction. Chromatin was captured by chromatin capture beads and then digested with a restriction enzyme. The end of the digest was filled in with biotin and ligated to form Intra-aggregated DNA. After cross-link reversal, 200 ng of DNA was sheared, using the covaris system. The sheared DNA fragments were repaired and ligated with Illumina adapters. The ligated DNA was purified, using Streptavidin Magnetic Beads and amplified to enrich the fragments. The quality of the amplified libraries was verified by capillary electrophoresis (Bioanalyzer, Agilent). Sequencing was performed, using an Illumina NovaSeq 6000 system, following provided protocols for 2 × 150 sequencing.

#### Genome *de novo* Assembly

*De novo* assembly was conducted, using FALCON-Unzip assembler with filtered subreads sequences. The length cut-off option was specified based on the subreads N50 value 23.8 kb. We performed error correction, using BWA version 0.7.10 and GATK version 3.5 with haplotig-merged primary contigs to improve the quality of genome assembly results. The dovetail Hi-C library was prepared according to the instructions of the manufacturer (Dovetail Hi-C Library kit). The hybrid scaffolding was performed, using HiRise software with Hi-C data to obtain assembly results at the pseudomolecule level. The assembly was assessed, using Benchmarking Universal Single-Copy Orthologs (BUSCO) (Simão et al., 2015).

#### Scaffold Anchoring Using Genetic Map

To construct the GBS library, genomic DNA of the ‘BS F_2_ (I)’ population extracted by the CTAB method (Lee et al., 2020) was double-digested, using EcoRI and MseI (Han et al., 2018). Subsequently, the adapters were ligated to both enzyme cut-site of digested gDNA with different barcodes for each sample. After amplification and quality control, the library generated was sequenced, using Illumina Hiseq 4000. The raw reads were aligned with the ‘assembly’, using the BWA v0.7.12. To group and sort the aligned read, Picard Tools v1.19 and SAMtools v1.1 were used. For SNP calling, the GATK Unified Genotyper v3.8-0 was used. SNPs were filtered for minimum genotype quality of Q30 and a minimum three-read depth. SNPs showing polymorphism between the two parental lines and segregated in the F_2_ population were used to genetic map construction.

To select the individuals for a single plate of Affymetrix of Axiom® Strawberry Genotyping Array (IStraw90K), the quality control (QC) analysis of the genomic DNA was performed, using ND-1000 spectrophotometer and Quant-iT™ PicoGreen® dsDNA Reagent and Kits. Among the high-quality gDNA, 94 individuals with higher recombination frequency (calculated based on GBS genotypes data) and the genotyping rate were selected one by one, and the Axiom SNP array experiment was performed at DNA Link, Seoul, Korea. The SNP array was based on the reference genome build of NCBI FAN_r1.1. The quality of each individual sample of raw data was determined, using the default dish quality control (DQC) values by Affymetrix Power Tools (APT) in the analysis workflow. After removing DQC, the genotype calling was performed, using APT with AxiomGT1 BRLMM-P algorithm. For genotyping analysis, Axiom Analysis Suite v4.0.1 was used. Only the markers classified into poly high resolution, a call rate below the threshold, and other categories were used for further analysis. To find the physical position of SNPs in the ‘Wongyo 3115’ genome, probe sequences of the array were aligned with ‘Wongyo 3115’ genome assembly. To construct a linkage map, the SNP markers obtained from GBS and Axiom analysis were used. Linkage analysis was performed using Carthagene software (De Givry et al., 2005), with a LOD threshold of 10.0 and a maximum distance of 30.0 cM (Han et al., 2016). To compare the physical and genetic maps, the flanking sequences of markers were aligned with the ‘Wongyo 3115’ assembly, using local blastn, using the Linux server (Maximum number of HSPs of 1.0 and maximum target sequence of 4.0). Only the markers with blast hits were visualized, using the ggplot2 package in R version 3.4.3 (RStudio, Boston, and USA).

#### Library Preparation and Iso-Seq Sequencing

The total RNA was isolated from the callus tissue, and the cDNA synthesis was carried out, using the SMARTer PCR cDNA Synthesis Kit (Clontech 634925) and PCR using PrimeSTAR GXL DNA Polymerase (Clontech R050A). Further purification was performed, using AMPure® PB Bead prior to the library construction. For the construction of SMRTbell library, 1-5 μg of pooled cDNA was prepared, using SMRTbell™ Template Prep Kit 1.0-SPv3 (PN 100-991-900). The SMRTbell library was sequenced, using SMRT cells per library (Pacific Biosciences, Sequel™ SMRT® Cell 1M v2). A total of four SMRT cells were sequenced, using the PacBio Sequel platform with 1,200 min of movie time.

### Gene Prediction and Annotation

Automated gene prediction was undertaken, using the automated annotation pipeline MAKER with default parameters (Stanke et al., 2006; Cantarel et al., 2008). Gene annotations were made, using all protein sequences of the *Fragaria* genus. *Ab initio* gene predictions were created by MAKER 2.31.8, using the programs SNAP 2006-07-28 and Augustus 3.2.3 (Korf, 2004; Holt and Yandell, 2011). Gene models were further improved by providing MAKER with the IsoSeq data generated. Post annotation was performed to add putative gene functions and protein domains, using BLAST to UniProt/Swiss-Prot and InteProScan v.85.0 (Jones et al., 2014). The Repeat modeler (http://www.repeatmasker.org/RepeatModeler/) and Repeat Masker 4.1.1 (Tarailo-Graovac and Chen, 2009) programs were employed to investigate the repeats in the ‘Wongyo 3115’ genome with the database search from DFAM and RepBase. Furthermore, annotation of tRNA was performed, using tRNAscan-SE43 software (Lowe and Eddy, 1997), with default parameters and rRNA annotation, using the Barrnap tool (github.com/tseemann/barrnap).

### Re-sequencing and Mapping

To assess the quality of our new reference sequence, re-sequencing and mapping of 10 strawberry cultivars (Supplementary Table 9) widely cultivated in Asia and USA were performed. The DNA libraries were prepared, using Illumina TruseqNano DNA HT Kit according to the protocol of the manufacturer. Initially, the extracted DNA was fragmented into indexed shotgun paired-end libraries (maximum 550 bp inserts), using Covaris M220 (Woburn, MA, USA). Subsequently, the fragments of DNA were repaired, adenylated, and adapter ligated before the size selection and amplification. Quality control was further carried out with the resulting DNA library, using an Agilent Technologies 2100 Bioanalyzer (Agilent Technologies) to analyze the size distribution of the DNA and to eliminate contamination. Finally, pair-end sequencing was performed with the Illumina Novaseq system, which produced maximum 16 Gb output data for each sample. Furthermore, quality control of removing the low-quality base of reads and adaptor sequences was performed, using each software, FastQC v.0.11.9 and Trimmomatic v.0.39. From quality control results, high-quality reads were mapped to ‘Wongyo 3115’ and ‘Camarosa’ genomes, using BWA (0.6.1-r104), with the following parameters: maximum number of gap extension (-e), 50; seed length (-l), 30; the maximum difference in the seed (-k), 1; number of threads (-t), 32; mismatch penalty (-M), 6; gap open penalty (-O), 15; and gap extension penalty (-E) was set to 8.

### Construction of the High-Density Linkage Map and QTL Analysis

A total of 186 ‘BS F_2_ (II)’ individuals were genotyped by Axiom® IStraw35 array (Verma et al., 2017a). Flanking sequences of markers were aligned with the ‘Wongyo 3115’ and ‘Camarosa’ genomes, using BLAST, and markers aligned with unassembled scaffolds were filtered out (Supplementary Table 8). Additionally, markers polymorphic between parental lines and the F_2_ populations were used for genetic map construction. All the markers except the markers nonsegregated in F_2_ (for example, A:H:B = 0:0:186 or A:H:B = 186:0:0) were used (Supplementary Dataset 3). The bin map was constructed by a sliding window approach with window size 20 flanking markers for chromosomal rearrangement detection (Han et al., 2016). For QTL analysis, linkage groups were generated, using the axiom markers, and each linkage group was assigned to the chromosomes, using BLAST results. After assigning chromosomes, a bin map was constructed by the sliding window approach with window size 2 Mbp. Linkage maps of bins were constructed, using Carthagene with LOD threshold 5.0 and distance threshold 30 cM (De Givry et al., 2005).

Firmness data evaluated in 2019 and 2020 were used for QTL analysis. Using Windows QTL Cartographer 2.5 (Wang et al., 2012), QTLs were detected by composite interval mapping with a default option. Significant QTLs were selected based on the LOD threshold calculated by the 500 times permutation test (*P*-value < 0.05). QTL regions were estimated by a 99% confidence interval of each QTL, and closely linked bins were used to predict physical position of the QTLs. The candidate genes were retrieved based on the physical position and the annotation data of ‘Wongyo 3115’.

### Comparative Mapping

The physical and genetic maps of the ‘Wongyo 3115’ and ‘Camarosa’ genomes were compared by the axiom markers aligned with both genomes commonly. In addition, the physical map of ‘Wongyo 3115’ was compared with previously reported SNP-based genetic maps (Sánchez-Sevilla et al., 2015; Lee et al., 2020; Alarfaj et al., 2021). Flanking sequences or primers were aligned with the ‘Wongyo 3115’ genome by BLAST. The single linkage group was assigned to the one chromosome that most number of the markers of the linkage groups was aligned. MapChart2.2 (Voorrips, 2002) was used to draw comparative maps.

### Transcriptome Sequencing and Expression Analysis

The achenes (skin) and receptacle (flesh) tissues were separated from matured fruit samples of ‘Wongyo 3115’ (a high-firm inbred line) and ‘P69’ (a low-firm inbred line), using a scalpel according to Sánchez-Sevilla et al. (2017). Three biological replicates were employed for transcriptome analysis. The samples were homogenized, and the total RNA was extracted, using Trizol (Invitrogen, USA) according to the protocol of the manufacturer. After the quality evaluation, cDNA libraries were prepared from the RNA samples, and the paired-end library was constructed, using the Truseq stranded mRNA Prep kit (Illumina) according to the instructions of the manufacturer. After purification, the sequencing library was produced by PCR amplification and sequenced, using the Novaseq6000 platform (Illumina). The raw reads with low quality and the clean reads were then assembled and mapped to the ‘Wongyo 3115’ reference genome, using the Top hat v2.0.13 (Trapnell et al., 2009). The differential expression was analyzed, using the cuffdiff v2.2.0 (Trapnell et al., 2010). Genes with the FPKM estimate were 2-fold higher than that of the lowest one and were identified as differentially expressed genes (DEGs). Gene expression differences were validated, using a chi-square test and false discovery rate (FDR). Genes with an FDR < 0.001 and for which the FPKM estimate was 2-fold higher than that of the lowest one were identified as DEGs. The functional annotations were performed, using DAVID 6.8 Beta. A heat map was generated, using significantly altered genes in fruits of both cultivars. The raw intensity data (FPKM) were log2 transformed and then utilized for the calculation of *Z* scores.

## Data Availability Statement

Whole-genome sequence data of ‘Wongyo 3115’ have been deposited in NCBI under the Bioproject PRJNA662854 and Biosample SAMN16094694 (accession number SRR14102268-SRR14102276). This whole genome shotgun project has been deposited at GenBank under the accession JACXYW000000000. The version described in this paper is JACXYW010000000. Re-sequencing data of 10 cultivars and RNA-Seq data have been deposited in NCBI under the Bioproject PRJNA727900 and PRJNA728506, respectively.

## Author Contributions

H-EL, AM, KH, D-SK, and B-CK designed the experiments. H-EL performed the sequencing part. AM performed the bioinformatics analysis and wrote the manuscript. KH constructed genetic map and performed QTL analysis. SL and IR developed the ‘Wongyo 3115’ homozygous line. JK assisted in the genome assembly and annotation. J-GY, JJ, and B-CK constructed the genetic linkage map and assisted in scaffold anchoring. JK, EL, and Y-RL assisted in the genome annotation and manuscript drafting. D-SK and B-CK managed the project and acquired funding. All the authors have proofread and finalized the manuscript.

## Conflict of Interest

The authors declare that the research was conducted in the absence of any commercial or financial relationships that could be construed as a potential conflict of interest.
