# Supplementary material for: Chromosome Level Assembly of Homozygous Inbred Line ‘Wongyo 3115’ Facilitates the Construction of a High-Density Linkage Map and Identification of QTLs Associated With Fruit Firmness in Octoploid Strawberry (Fragaria × ananassa)
Source: Front Plant Sci. 2021 Jul 14;12:696229. doi: 10.3389/fpls.2021.696229 (PMC8317996; doi:10.3389/fpls.2021.696229)
Supplement: Supplementary file 6 [file Data_Sheet_6.pdf]

Chromosome level assembly of homozygous inbred line 'Wongyo 3115' facilitate the construction of high density linkage map and identification of QTLs associated with fruit firmness in octoploid strawberry (*Fragaria* × *ananassa*)

Supplementary Tables

Supplementary Table 1. Summary of Illumina short read sequencing 'Wongyo 3115'

Read Order	Index	Yield(Bases)	Reads	% of >= Q30	Mean Quality
1	CAGATC	17,717,438,180	175,420,180	83.67	34.11
2	CAGATC	17,717,438,180	175,420,180	75.31	32.33

Supplementary Table 2. Genome size estimation using JELLYFISH

k-mer	17	19	25
Total Nucleotides	26,021,674,618	25,242,267,482	23,339,394,113
Peak Depth	33	32	29
Genome Size	788,535,594	788,820,859	804,806,694

Supplementary Table 3. Summary of PacBio filtered subreads

No. of Bases	No. of Reads	N50 Read Length	Mean Read Length	Max Read Length
61,006,607,136	4,132,073	23,811	14,764	99,891

Supplementary Table 4. Summary of FALCON-Unzip assembly statistics.

	draft		unzip		arrow		Error correction
	primary	associated	primary	haplotig	primary	haplotig	Primary
Number of contigs	1,296	102	365	918	323	844	323
Total size of contigs	812,765,282	12,033,549	803,308,313	62,138,683	805,586,201	59,823,691	805,664,111
Longest contig size	22,743,024	861,039	22,759,814	908,834	22,877,306	916,648	22,878,989
Number of contigs > 1K nt	1,251	101	364	918	323	844	323
No.of contigs > 10K nt	708	99	364	907	323	839	323
No.of contigs > 100K nt	176	27	175	102	173	103	173
No.of contigs > 1M nt	122	0	122	0	122	0	122
No. of contigs > 10M nt	26	0	26	0	26	0	26
Mean contig size	627,134	117,976	2,200,845	67,689	2,494,075	70,881	2,494,316
Median contig size	11,244	59,840	88,105	53,007	158,413	54,930	158,413
N50 contig length	9,657,506	256,692	9,658,731	73,343	9,848,239	75,523	9,849,455
L50 contig count	30	13	30	234	29	217	29
GC Contents (%)	39.47	39.78	39.46	42.51	39.36	42.19	39.35

Supplementary Table 5. Dovetail-HiRise comparative assembly statistics

**Parameters**	**Input assembly**	**Dovetail HiRise assembly**
Total Length	805.66 Mb	805.68 Mb
L50/N50	29 scaffolds; 9.849 Mb	13 scaffolds; 27.373 Mb
L90/N90	89 scaffolds; 2.435 Mb	26 scaffolds; 21.949 Mb
Longest Scaffold	22,878,989 bp	38,443,702 bp
Number of scaffolds	323	208
Number of gaps	0	135
Number of breaks made to input assembly by HiRise	13	
Number of joins made by HiRise	133	
Library statistic	103M read pairs; 2x151 bp	

Supplementary Table 6. Evidence sources employed for gene prediction

**Sample Source**	**Data Source**	**Number of Sequences**
'Wongyo 3115'	IsoSeq	108,951
Fragaria iinumae	Protein	76,760
Fragaria nipponica	Protein	87,803
Fragaria nubicola	Protein	85,062
Fragaria orientalis	Protein	99,674
Fragaria vesca	Protein	28,588

Supplementary Table 7. Summary of transposons identified in 'Wongyo 3115'genome

**Repeats**	**Number of elements**	**Length (bp)**	**Percentage of sequence (%)**
SINEs	11,672	15,32,580	0.19
LINEs	30,854	11,626,257	1.44
LTR elements	1,57,093	1,55,181,296	19.26
DNA elements	1,87,134	87,941,340	10.92
Unclassified	2,02,708	43,831,916	5.44
Small RNA	13,085	31,759,51	0.39
Satellites	1,013	5,42,848	0.07
Simple repeats	2,05,878	8,72,0425	1.08
Low complexity	35,429	1,768,696	0.22

Supplementary Table 8. BUSCO analysis of strawberry genome

**BUSCO terms**	**Embryophyta (n: 1,440)**	**Eukryota (n: 303)**
Complete BUSCOs	1,355 (94.1%)	274 (90.4%)
Complete and single-copy BUSCOs	111 (7.7%)	27 (8.9%)
Complete and duplicated BUSCOs	1,244 (86.4%)	247 (81.5%)
Fragmented BUSCOs	11 (0.8%)	2 (0.7%)

Supplementary Table 9. Comparison of re-sequencing mapping ratio of strawberry cultivars with 'Wongyo 3115' and 'Camarosa' reference genomes.

	**Re-sequencing data**		**^{z} Percentage of mapped reads (%)**		**^{y} Genome coverage (%)**	
**Cultivars**	**Origin**	**No. of total reads**	**'Wongyo 3115'**	**'Camarosa'**	**'Wongyo 3115'**	**'Camarosa'**
Chandler	USA	92,994,730	90.05	87.84	92.70	93.48
Comet	USA	97,763,002	89.85	87.12	92.29	91.94
Diamante	USA	100,102,696	91.04	88.70	92.26	93.67
Elsanta	Europe	95,387,670	88.01	85.53	91.84	92.33
Florida_belle	USA	96,049,834	88.42	85.33	91.51	91.77
Gaviota	Europe	98,395,888	90.44	89.17	92.27	93.65
General_Chanzy	Asia	93,486,600	90.10	87.84	92.42	92.25
Misohyang	Asia	95,758,136	91.66	87.33	85.81	91.26
Sure_crop	USA	97,045,784	88.51	85.86	93.30	80.08
Ventana	USA	90,137,424	90.44	90.14	91.66	89.70
**Average**		**95,712,176**	**89.85%**	**87.49%**	**91.61%**	**91.01%**

^{z} Percentage of mapped reads = Number of reads mapped/ Total number of reads
^{y} Genome coverage = Percentage of mapped region / Reference genome size

Supplementary Table 10. Summary of Axiom 35K markers and the filtering criteria employed for the genetic map construction.

**Filtering option**	**Number of markers**
Raw marker	37,996
Polymorphic marker b/w parental lines	6,494
Aligned to chromosome (identity > 90%)	5,527
Segregated in F_2*	
Aligned to one chromosomal region	4,466
Or linked to LG matched to one chromosome	

*Markers with all maternal, paternal, or heterozygous genotype were removed.

Supplementary Figures

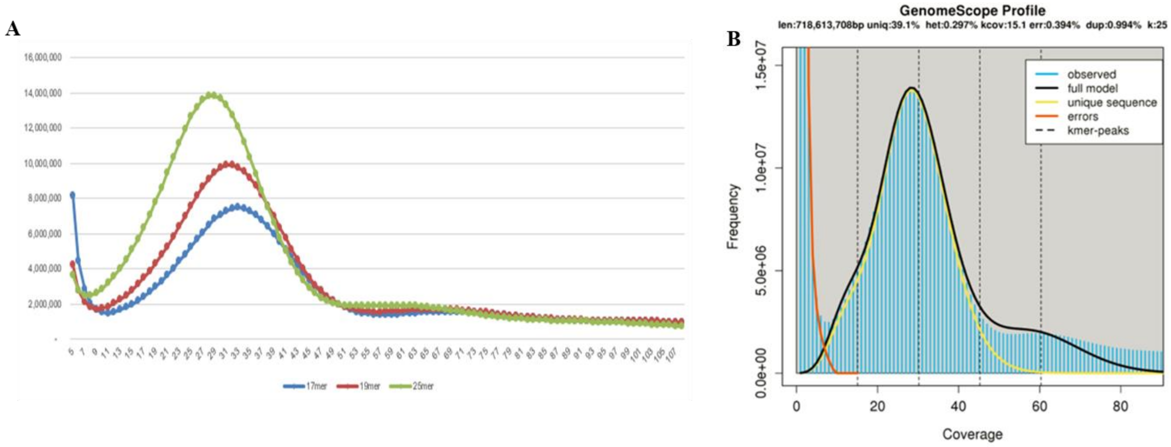

Supplementary Fig. 1. Estimation of genome size. A) K-mer based estimation of ‘Wongyo 3115’ genome size. B) Genome heterozygosity prediction based on GENOMESCOPE program.

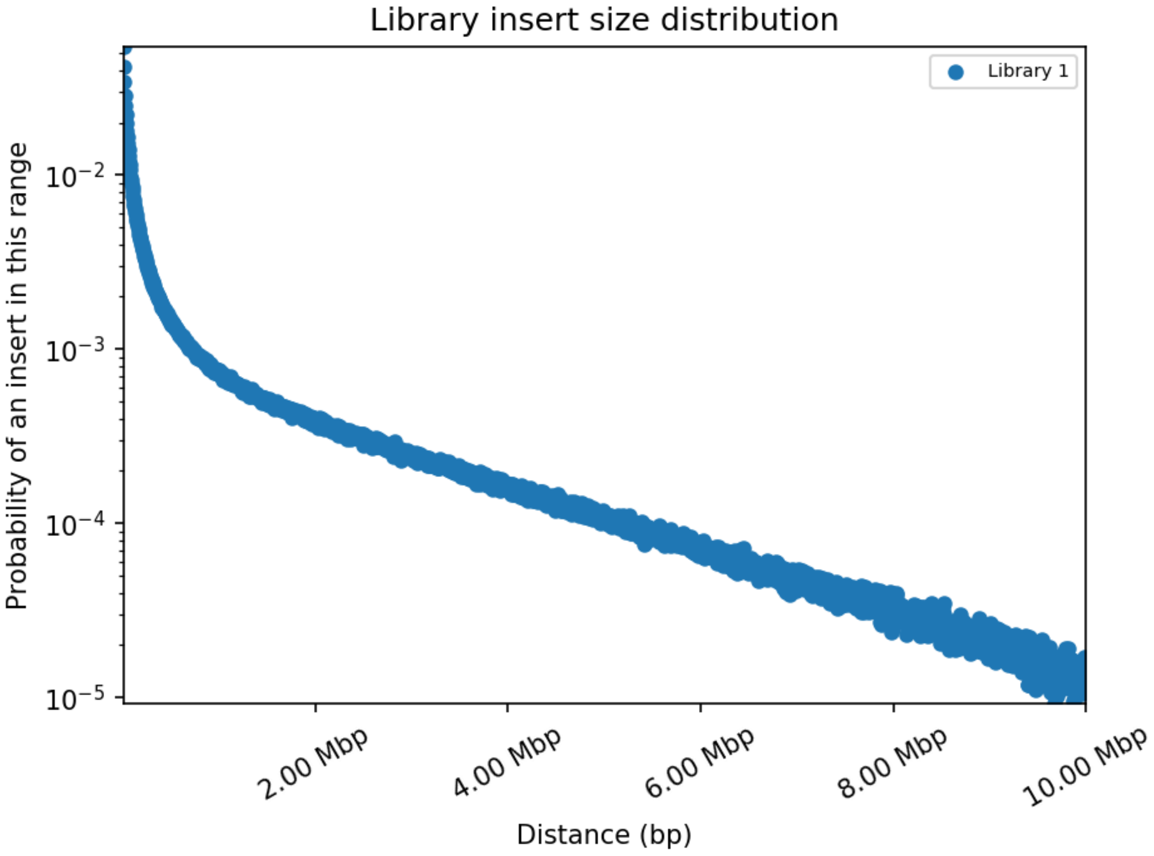

Supplementary Fig. 2. Distribution of insert sizes in the Dovetail library. The distance between the forward and reverse reads is given on the X-axis in basepairs, and the probability of observing a read pair with a given insert size is shown on the Yaxis.

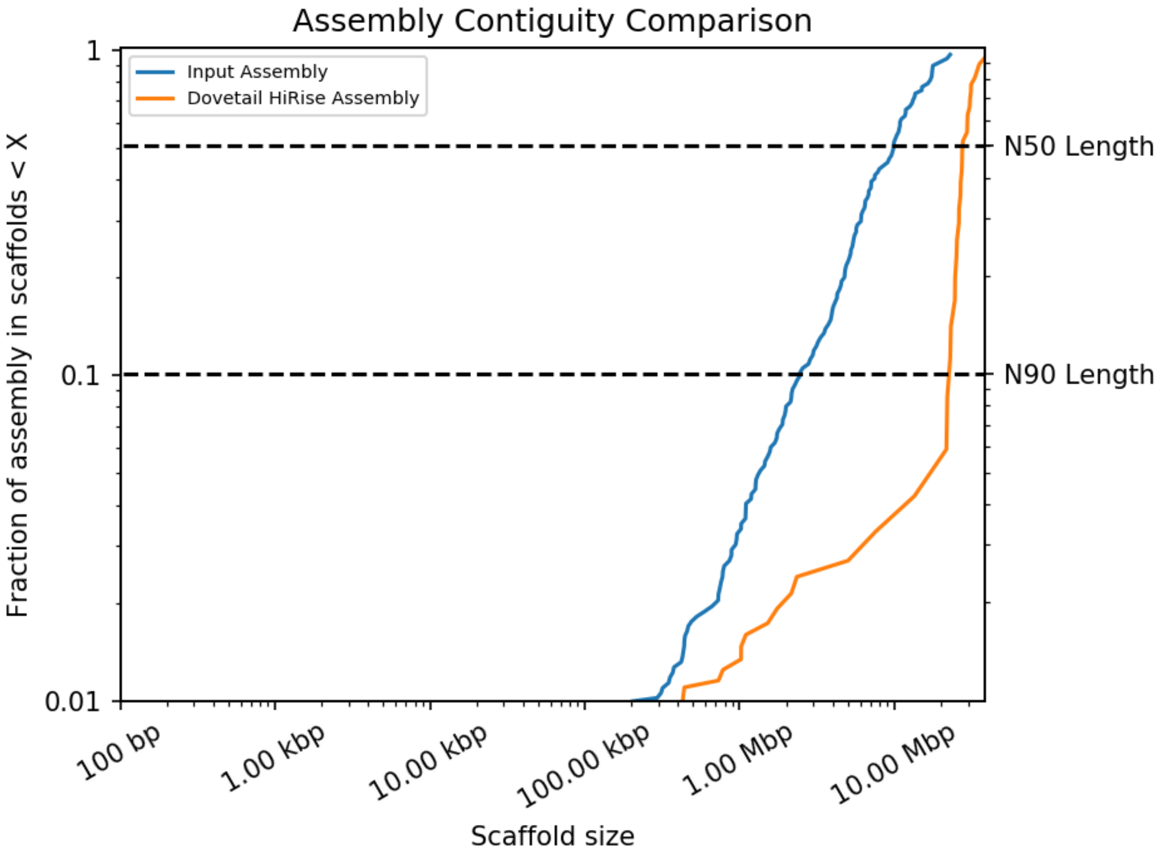

Supplementary Fig. 3. A comparison of the contiguity of the input assembly and the final HiRise scaffolds. Each curve shows the fraction of the total length of the assembly present in scaffolds of a given length or smaller. The fraction of the assembly is indicated on the Y-axis and the scaffold length in basepairs is given on the X-axis. The two dashed lines mark the N50 and N90 lengths of each assembly. Scaffolds less than 1 kb are excluded.

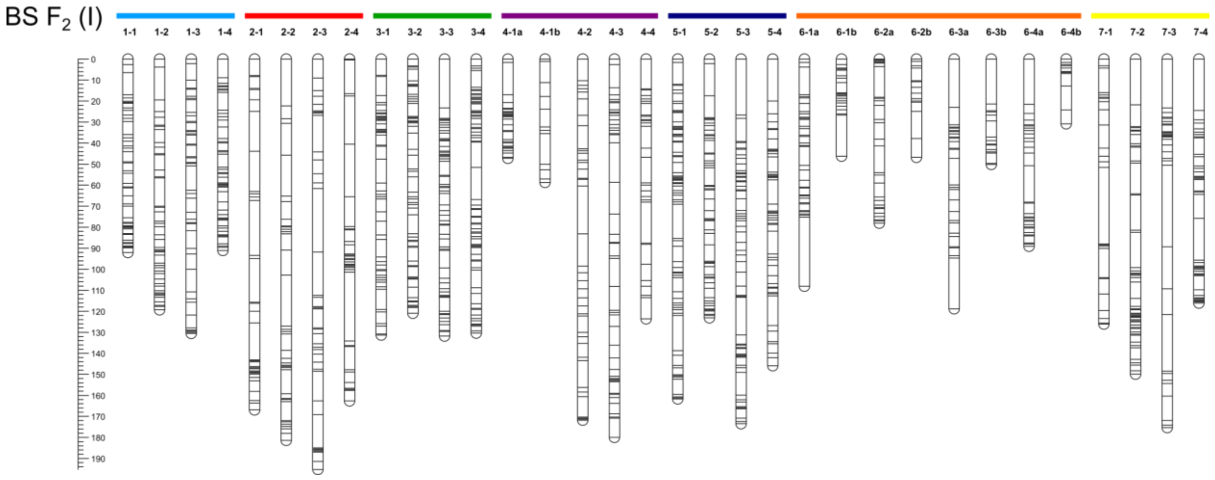

Supplementary Fig.4. Construction of 'BS F_2 (I)' linkage bin map**.** High-density linkage bin map of the 'BS F_2 (I)' population consists of GBS and Axiom 90K marker sets. Linkage groups are denoted corresponding to 28 chromosomes of 'Wongyo 3115'. Chr4-1, 6-1, 6-2, 6-3 and 6-4 are divided into two linkage groups. The bar on the left is an indicator of the length of the linkage groups.

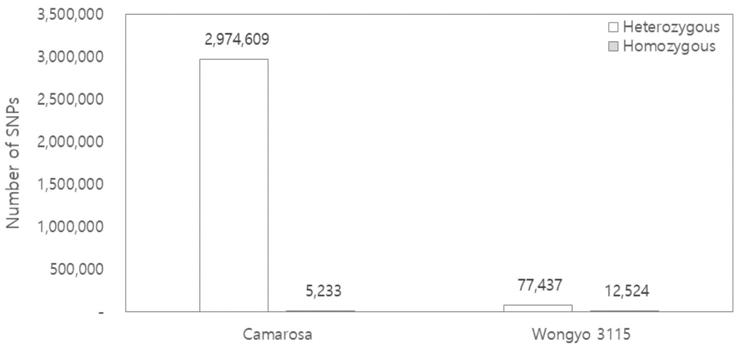

Supplementary Fig.5. Number of SNPs detected utilizing the Illumina sequencing data in 'Camarosa' and 'Wongyo 3115' genomes representing the high homozygosity of 'Wongyo 3115'.

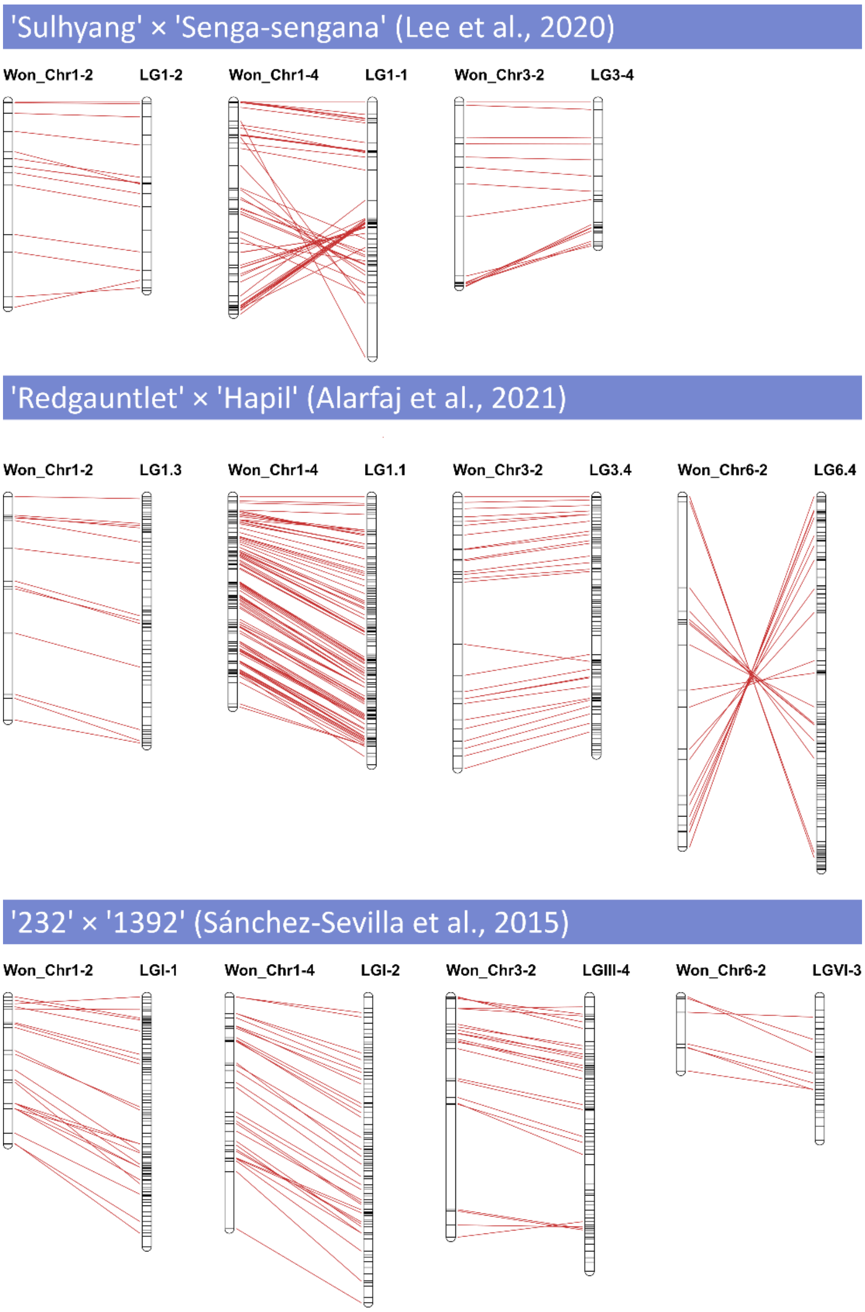

Supplementary Fig. 6. Comparison of previously reported genetic maps (Lee et al., 2020; Alarfaj et al., 2021; Sánchez-Sevilla et al., 2015) and physical map of chromosome 1-2, 1-4, 3-2, and 6-2 of 'Wongyo 3115'.

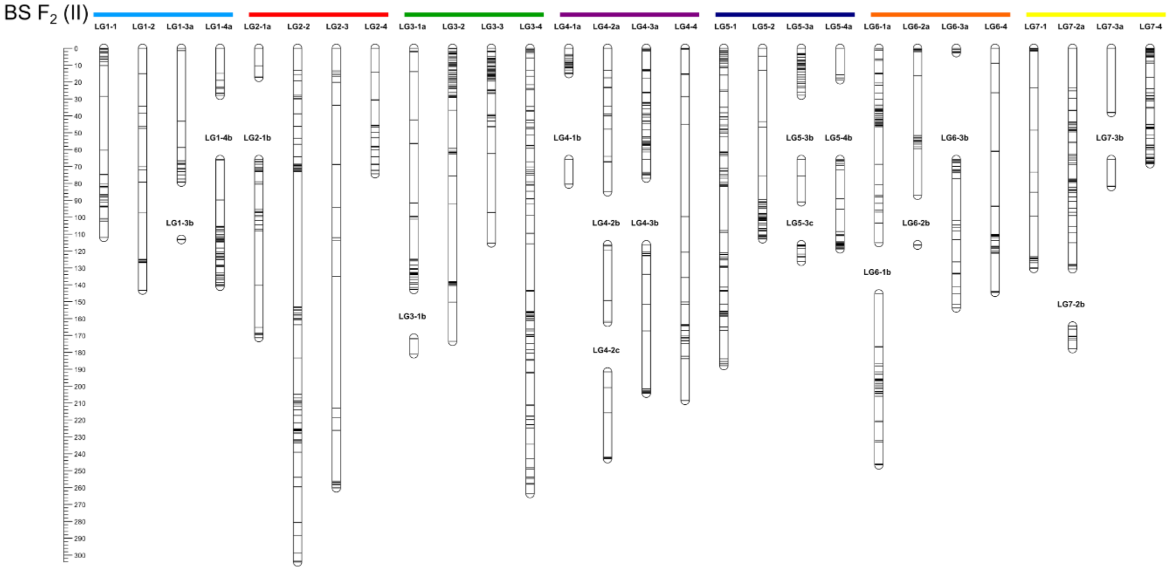

Supplementary Fig. 7. Linkage map of 'BS F_2 (II)' used for QTL analysis

Year	2004	2005		2006		2007		2008		2009	2010 - 2012	
Selfing generation(Sn)	Origin	S_1	S_2	S_3	S_4	S_5	S_6	S_7	S_8	S_9	S_{10}	
		1	1	1	1	1	1	1	1	1		
		:	:	:	:	:	:	:	:	:		
	Benihoppe	3 →	5 →	1 →<br>2 →	8 →<br>10→	307→<br>72 →	8 →<br>8 →	138→<br>46 →	5 →<br>5 →	5<br>14→	3	**(B8-9)**<br>**(P69)**
		:	:	:	:	:	:	:	:	:		
(No. of breeding lines)		258	346	112	220	320	40	140	40	40	40	

**Sn**	**B**	**S1**	**S2**	**S3**	**S4**	**S5**	**S6**	**S7**	**S8**	**S9-10**
Homozygosity	50	→								90-100

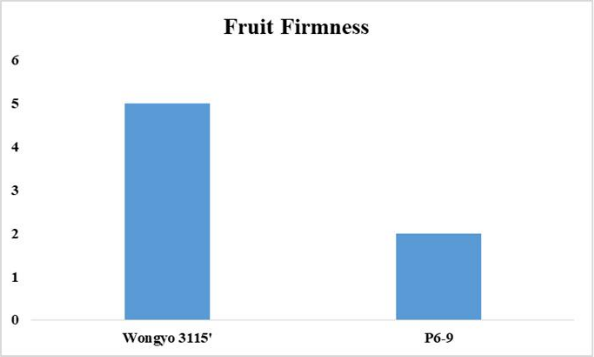

'Wongyo 3115 '

P6-9

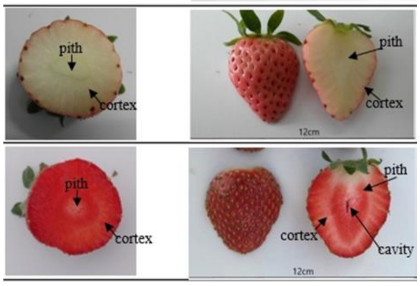

Supplementary Fig. 8. Pedigree and homozygosity details of 'Wongyo 3115' (B8-9) and 'P69' inbred lines along with the phenotype of fruits in 'Wongyo 3115' and 'P69' inbred lines.

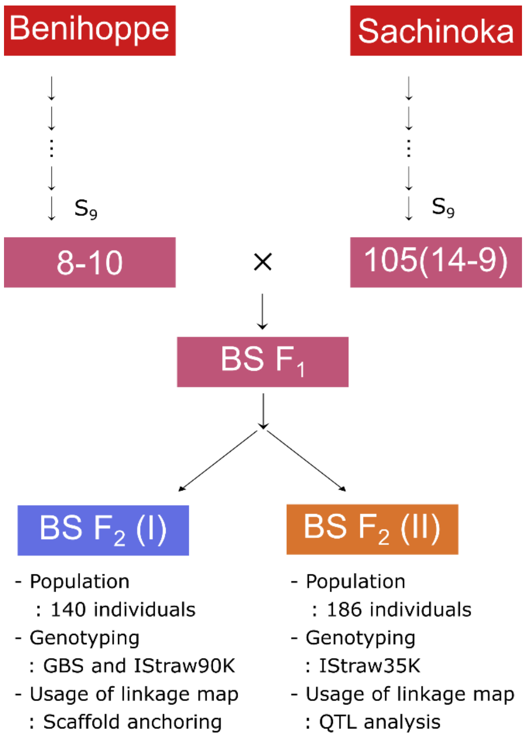

Supplementary Fig. 9. A pedigree diagram of two BS F_2 populations.
